# Plant Long Noncoding RNAs: New Players in the Field of Post-Transcriptional Regulations

**DOI:** 10.3390/ncrna7010012

**Published:** 2021-02-17

**Authors:** Camille Fonouni-Farde, Federico Ariel, Martin Crespi

**Affiliations:** 1 Université Paris-Saclay, CNRS, INRAE, Univ Evry, Institute of Plant Sciences Paris-Saclay (IPS2), Bat 630, 91192 Gif sur Yvette, France; camille.fonouni-farde@universite-paris-saclay.fr; 2 Université de Paris, CNRS, INRAE, Institute of Plant Sciences Paris-Saclay (IPS2), Bat 630, 91192 Gif sur Yvette, France; 3 Instituto de Agrobiotecnología del Litoral, CONICET, Universidad Nacional del Litoral, Colectora Ruta Nacional 168 km 0, 3000 Santa Fe, Argentina; fariel@santafe-conicet.gov.ar

**Keywords:** long noncoding RNA, post-transcriptional regulation, target mimicry, alternative splicing, protein re-localization, translation promotion, post-translational modification

## Abstract

The first reference to the “C-value paradox” reported an apparent imbalance between organismal genome size and morphological complexity. Since then, next-generation sequencing has revolutionized genomic research and revealed that eukaryotic transcriptomes contain a large fraction of non-protein-coding components. Eukaryotic genomes are pervasively transcribed and noncoding regions give rise to a plethora of noncoding RNAs with undeniable biological functions. Among them, long noncoding RNAs (lncRNAs) seem to represent a new layer of gene expression regulation, participating in a wide range of molecular mechanisms at the transcriptional and post-transcriptional levels. In addition to their role in epigenetic regulation, plant lncRNAs have been associated with the degradation of complementary RNAs, the regulation of alternative splicing, protein sub-cellular localization, the promotion of translation and protein post-translational modifications. In this review, we report and integrate numerous and complex mechanisms through which long noncoding transcripts regulate post-transcriptional gene expression in plants.

## 1. Introduction

Unlike in prokaryotes, genomes in eukaryotes exhibit a large variability in their size [1,2], which does not always correlate with the number of protein-coding genes nor the developmental complexity of organisms. This paradox of an apparent imbalance between organismal genome size and morphological complexity, dubbed the “C-value paradox” [3,4], was in part solved by the extraordinary progress made in next-generation sequencing technologies. Indeed, eukaryotic transcriptomes include a large fraction of non-protein-coding components [5]. Although up to 90% of eukaryotic genomes is estimated to be transcribed during development, only an estimated 2% of transcribed RNAs will code for proteins [6,7]. The noncoding genome, long considered silent and declared as “junk DNA” due to its high content in pseudogenes, simple repeats, and transposons [8,9], encodes a plethora of noncoding RNAs (ncRNAs) with unarguable biological functions. These comprise housekeeping RNAs (small nuclear and nucleolar RNAs, transfer RNAs, ribosomal RNAs, telomerase RNAs, tRNA-derived fragments, and tRNA halves), small regulatory RNAs (micro RNAs, small interfering RNAs, piwi-interacting RNAs, and Y RNAs), and long noncoding RNAs (lncRNAs), also including enhancer RNAs, transposon-derived RNAs, and circular RNAs [10].

LncRNAs form the most diversified group of ncRNAs, exhibiting a large range of sizes varying from 200 bases to over 100 kb in length. They are expressed in various tissues, cell-types, and cell-states, and function in the nucleus or cytoplasm [11,12]. Given their vast diversity, lncRNAs are commonly classified according to their location and orientation relative to neighboring protein-coding transcripts. Long intronic RNAs are transcribed exclusively from intronic regions, whereas long intergenic ncRNAs lie outside of genes and include promoter-, enhancer-, and transposable element-derived lncRNAs and sometimes give rise to double-stranded RNAs. Sense and antisense double-stranded lncRNAs are transcribed from the sense and antisense strands, respectively, while natural antisense transcripts (NATs) initiate in the reverse strand of sense protein coding regions (*cis*-NATs) or are complementary to a sense transcript located in a distinct genomic locus (*trans*-NATs) [6,13]. CircRNAs constitute a novel class of lncRNAs consisting in covalently closed molecules of single-stranded RNA, resulting from back-splicing, a non-canonical form of alternative splicing [14]. Alternatively, lncRNAs can be further categorized depending on their molecular functions and interactions with additional regulatory molecules such as proteins, DNA, or other RNAs [15,16].

It is increasingly clear that lncRNAs participate in virtually every aspect of gene expression. In plants, although the functional characterization of ncRNAs is still in its early stages, several lncRNAs have been described as regulators of gene transcription, capable of conditioning the epigenetic environment of their genomic targets and of modulating the activity of transcriptional complexes [17]. In addition, at the post-transcriptional level, various lncRNAs have been associated with complementary target-RNA degradation, alternative splicing, promotion of translation, protein sub-cellular localization and post-translational modifications. Notably, lncRNA-mediated post-translational modifications of histones are related to the transcriptional regulation of target genes, which has been recently reviewed [17]. Here, we report and integrate recent discoveries about plant lncRNA-mediated regulations of post-transcriptional gene expression.

## 2. Long Noncoding RNAs Mediating Complementary Target-RNA Degradation 

**Figure 1:**
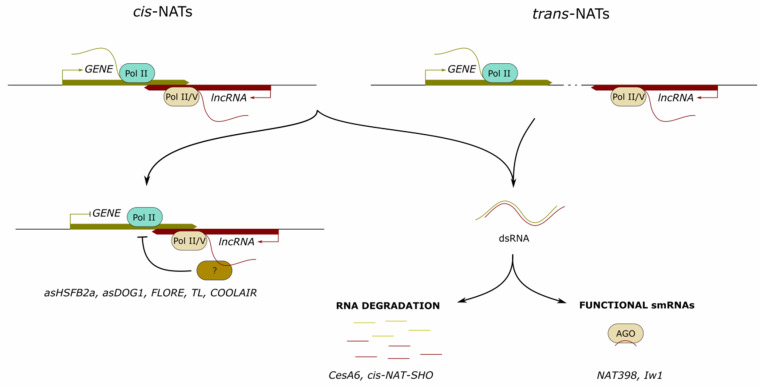
Long noncoding RNAs forming RNA–RNA pairs in the nucleus. Long noncoding (lnc) RNAs can form RNA pairs (dsRNA) with complementary mRNAs in *cis* (*cis*-NATs) or in *trans* (*trans*-NATs), leading to RNA degradation or to the formation of functional small (sm) RNAs. In addition, antisense transcripts can locally recruit protein partners that modulate the transcriptional activity of overlapping protein-coding genes. Examples of characterized lncRNAs are indicated at the bottom.

Natural antisense transcripts (NATs) constitute an important class of lncRNAs, exerting a wide variety of molecular functions in eukaryotes [18,19]. They are complementary to sense mRNAs and can be classified into *cis*-NATs generated from a single locus showing sequence complementarity with their corresponding sense transcript or *trans*-NATs that are transcribed from different distant loci and typically display imperfect complementarities with their target endogenous RNA [20,21] (Figure 1). In silico analyses performed in several plant species have led to the identification of a large number of NATs [20,22,23,24,25,26]. In particular, a genome-wide analysis using a custom-designed NAT array revealed that up to 70% of annotated mRNAs have complementary NATs in *Arabidopsis thaliana* [27].

### 2.1. LncRNAs Involved in Discordant Regulation

NATs can affect positively (concordant regulation) or negatively (discordant regulation) the expression of sense transcripts. An example of discordant regulation is provided by the NAT-lncRNA *asHSFB2a* which counteracts the expression of the *HEAT SHOCK FACTOR B2a* (*HSFB2a*) mRNA in *A. thaliana* female gametophytes [28]. The overexpression of *asHSFB2a* in transgenic plants leads to the absence of *HSFB2a* RNA while the overexpression of *HSFB2a* results in a complete loss of *asHSFB2a* expression, suggesting that *HSFB2a* and *asHSFB2a* are mutually repressive [28]. Similarly, the NAT-lncRNA *DELAY OF GERMINATION 1* (*asDOG1*) was found to be a negative regulator of *DOG1* expression, a gene involved in the control of germination [29]. Down-regulation of *asDOG1* transcription increases the levels of *DOG1* sense mRNAs, enhancing seed dormancy [29]. As a last example in *A. thaliana*, a screening of lncRNAs using a custom-made array led to the identification of the circadian-regulated lncRNA *CDF5 LONG NONCODING RNA* (*FLORE*), a NAT of the *CYCLING DOF FACTOR 5* (*CDF5*) transcript, which likely connects the circadian clock to the photoperiodic flowering pathway [30]. *FLORE* is specifically expressed in the vasculature and regulates *CDF5* in *cis* as well as *CDF1* and *CDF3* in *trans*. Interestingly, *FLORE* and *CDF5* show a mutual inhibition behavior, suggesting that the *CDF5/FLORE* NAT pair constitutes a circadian regulatory module, which buffers its own circadian oscillation and photoperiodic flowering [30]. In rice, the NAT-lncRNA *TWISTED LEAF* (*TL*) is transcribed from the opposite strand of the *OsMYB60* locus encoding an R2R3 MYB transcription factor [31]. Downregulation of *TL* by RNA interference leads to a significant increase in *OsMYB60* expression levels and twisted leaf blades. It was suggested that *TL* may play a *cis*-regulatory role on *OsMYB60* by affecting H3K27me3, H3K36me2, and H3K36me3 histone mark deposition [31]. 

### 2.2. LncRNAs as Precursors of Small Regulatory RNAs

In addition, various NAT-lncRNAs have been reported to cause post-transcriptional silencing through the production of regulatory small interfering (si) RNAs derived from NAT pairs. This mechanism was first described in the regulation of salt tolerance in *A. thaliana* [32]. Under salt stress, the induction of *SRO5* mRNA allows the production of 24 nucleotides (nt) siRNAs from the region overlapping with Δ^1^-pyrroline-5-carboxylate dehydrogenase (*P5CDH*) transcripts. Subsequently, *P5CDH* transcripts are cleaved to generate 21nt siRNAs [32]. Similar mechanisms have been described in plant responses to pathogens and sperm cell development [33,34]. In barley, an increase in the NAT-lncRNA *CesA6* transcript levels leads to the production of 21 and 24nt siRNAs that correlates with the down-regulation of *CesA6* gene and several loci in *trans* involved in the regulation of cellulose rates and in the modulation of cell wall biosynthesis [35]. Similarly, in *Petunia hybrida*, the *Sho* gene involved in the production of cytokinin phytohormones contains an antisense ORF partially overlapping with the ORF of the *Sho* sense transcript that encodes the SHO protein [36]. The tissue specific transcription of *cis*-NAT *SHO* leads to the association of *Sho* sense and antisense transcripts in a double-stranded RNA likely targeted by a DICER complex for degradation into 24nt siRNAs [36]. Another mechanism, related to thermotolerance, was reported in *A. thaliana*. The NAT-lncRNAs *NAT398b* and *NAT398c* are *cis*-NATs of the *MIRNA* genes *MIR398b* and *MIR398c*, respectively. Knock-down of *NAT398b/c* promotes the accumulation of *MIR398b* and *MIR398c*, while the overexpression of *NAT398b* and *NAT398c* represses the processing of *miR398*. Notably, the overexpression of siRNAs derived from *NAT398* overlapping transcripts, so-called nat-siR398, reduces the levels of pri-miR398b and pri-miR398c [37].

Interestingly, computational analyses performed for *A. thaliana* revealed that antisense transcription is associated with micro (mi) RNA-targeted mRNAs [38]. In wheat, the lncRNA *INHIBITOR of WAX1* (*Iw1*) contains an inverted repeat showing more than 80% identity to the *WAX1-COE* gene, encoding a carboxylesterase-like protein that controls glaucousness [39]. The *Iw1* transcript is able to form a miRNA precursor-like long hairpin which produces small RNAs, including the 21nt-miRNA miRW1. The accumulation of miRW1 is linked to the down-regulation of *W1-COE* and its paralog *W2-COE*, the cleavage of *W1-COE* transcripts and glaucous repression [39].

## 3. Long Noncoding RNAs Involved in the Regulation of Alternative Splicing

**Figure 2:**
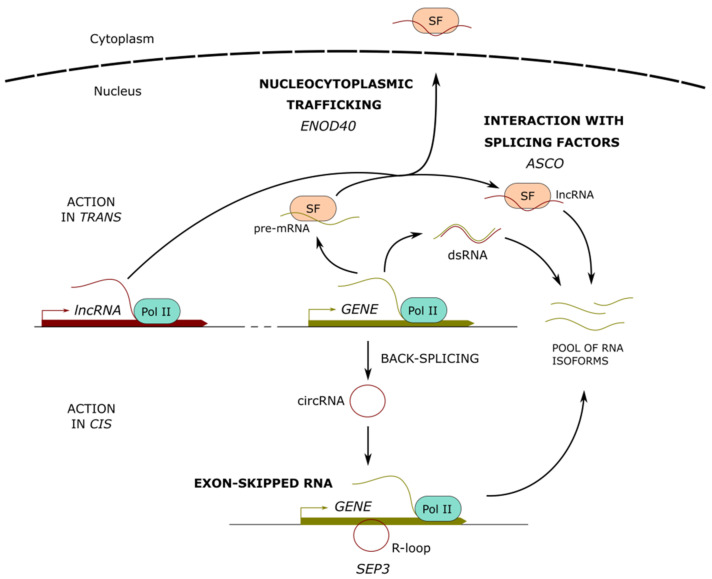
Long noncoding RNAs modulating alternative splicing. Long noncoding (lnc) RNAs can form RNA interactions (dsRNA) with pre-mRNAs, fine-tuning their splicing output. In addition, lncRNAs can interact with splicing factors (SF), affecting their recognition of pre-mRNA targets or their sub-cellular localization. Protein-coding transcripts can suffer back-splicing, leading to the formation of circular RNAs (circRNA), which can interact with the parent gene to form DNA-RNA duplexes (R-loops) and modulate the alternative splicing of the nascent transcripts. Examples of characterized lncRNAs are indicated below each mechanism.

In addition to capping and polyadenylation, the production of mature mRNAs from pre-mRNAs relies on the prior removal of introns and the ligation of the majority of exons in the order in which they appear in a gene, a process known as RNA splicing. Under certain circumstances, some exons can be skipped, generating various isoforms of mature mRNAs from a single pre-mRNA. This process, called alternative splicing (AS), is mediated by the spliceosome and involves a subclass of small nuclear RNAs referred to as nuclear uridine-rich RNAs. They function in collaboration with core small nuclear ribonucleoprotein complex subunits (snRNPs) and non-snRNPs splicing factors (SFs) whose interaction with lncRNAs likely condition their stability and sub-cellular localization [40,41,42,43]. LncRNAs mainly regulate AS through interactions with specific SFs, by the regulation of chromatin remodeling that fine-tunes the splicing of specific targets and via the formation of lncRNA-pre-mRNA duplexes [43] (Figure 2).

### 3.1. LncRNAs Interacting with Splicing Factors

In plants, AS plays crucial functions in the control of gene expression, boosting the protein-coding capacity and contributing to developmental plasticity [44,45]. In Arabidopsis, the lncRNA *ALTERNATIVE SPLICING COMPETITOR (ASCO)* interacts in vivo with the plant-specific SFs NUCLEAR SPECKLE RNA-BINDING PROTEINS (NSRa and b), which localize in nuclear speckles and are involved in splicing [46]. NSRs participate in the regulation of molecular and growth responses to auxin. After an auxin treatment, the double mutant *nsra/b* exhibited over 2200 genes differentially regulated in comparison to wild-type plants, as well as a reduced number of lateral roots suggesting a decreased sensitivity to auxin. Interestingly, the identification of RNA processing events in the *nsra/b* mutant revealed an important number of intron retention events and differential 5′ start or 3′ ends in a subset of genes, including a high number of auxin-related genes that behave accordingly in the *ASCO* overexpressing lines [46,47]. Remarkably, in vitro binding assays additionally showed that *ASCO* competes with mRNA-targets for the binding to NSRs, suggesting that *ASCO* regulates the AS of pre-mRNAs in response to auxin by hijacking NSRs [46]. More recently, a NSRa-directed RNA immunoprecipitation (RIP)-Seq approach in *A. thaliana* revealed that lncRNAs are overrepresented among NSRa targets [48]. As NSRa targets are mainly enriched for genes related to biotic stress responses, the interplay between lncRNAs and AS mRNAs in NSR-containing complexes was suggested to integrate the auxin and immune response pathways [48]. In agreement with this expectation, both knock-down and overexpression of *ASCO* led to the deregulation of expression and splicing of a large number of genes related to biotic stress and flagellin response in *A. thaliana* [49]. Remarkably, RNAi-*ASCO* plants and the double mutant *nsra/b* were found to exhibit a different response to flagellin, suggesting that *ASCO* also modulates AS in an NSR-independent manner. Consistently, an *ASCO*-directed chromatin isolation by RNA purification (ChIRP) coupled to mass spectrometry and RIP assays allowed the identification of other putative *ASCO* protein partners, including the pre-mRNA-processing-splicing factor 8A (PRP8a) and the spliceosome-core component SmD1b [49,50,51]. As previously observed for NSRs [46], *ASCO* overexpression also competes for PRP8a binding to particular mRNA targets [49].

### 3.2. LncRNAs Regulating Splicing Through Chromatin Remodeling

An additional mechanism of AS regulation involving circular non-coding RNAs (circRNAs) was reported in *A. thaliana* [52]. The overexpression of a circRNA comprising the entire exon 6 of the *SEPALLATA 3* (*SEP3*) gene increases the abundance of the naturally occurring exon-skipped AS variant *SEP3.3*, which lacks exon 6 [52]. SEP3 is a member of the plant MADS (MCM1-AGAMOUS-DEFICIENS-SRF)-box transcription factor superfamily involved in flower development, and modifications of *SEP3* splicing gives rise to floral homeotic phenotypes [52,53]. Remarkably, *SEP3* exon 6 circRNA can directly interact with its cognate DNA locus, forming an RNA:DNA hybrid (R-loop), which results in transcriptional pausing and correlates with the recruitment of splicing factors and AS. This mechanism suggests that circRNAs expressed from distant loci may increase the splicing efficiency of their cognate exon-skipped messenger RNAs and that chromatin conformation and R-loop formation are critical modulators of splicing patterns [52].

### 3.3. LncRNA-RNA Duplexes Regulating Alternative Splicing

The analysis of transcription data for overlapping gene pairs in *A. thaliana* revealed a large proportion of convergently overlapping pairs (COPs) with the potential to form double-stranded RNAs [23]. Interestingly, intron-containing genes and genes with alternatively spliced transcripts are over-represented among COPs. In addition, the loci where antisense transcripts overlap with sense transcript introns mostly show AS and/or variation of polyadenylation, suggesting that the formation of NAT lncRNA-RNA pairs may regulate the AS of protein-coding genes [23]. Consistently, a genome-wide screen of *trans*-NATs in Arabidopsis led to the identification of 1320 putative *trans*-NAT pairs [24]. Most of them are predicted to form extended double-stranded RNA duplexes if sense and anti-sense are expressed in the same sub-cellular compartment, and they may lead to gene silencing and indirect AS regulation [24]. Taken together, these studies suggest that lncRNAs integrate a dynamic splicing network to control transcriptome reprogramming through AS.

## 4. Long Noncoding RNAs as Molecular Cargos for Protein Re-Localization

Short open reading frame (sORF) mRNAs are atypical mRNAs that contain only sORFs (shorter than 100 amino acids) and accumulate in the cytoplasm where they can be translated into oligopeptides acting as signal molecules [54,55]. Remarkably, in legumes, the highly conserved *EARLY NODULIN 40* (*ENOD40*) genes known to participate in root symbiotic nodule organogenesis, contain only sORFs whose transcripts may encode short peptides [56,57,58]. In soybean, the lncRNA *GmENOD40* encodes two oligopeptides of 12 and 24 aa residues that may have a transport function and specifically bind to sucrose synthase subunit nodulin 100 to control the use of sucrose in nitrogen-fixing nodules [58]. In *M. truncatula*, *MtENOD40* is rapidly induced by symbiotic rhizobial bacteria in the root pericycle and is also detected in the differentiating cells of the nodule primordia [56,57]. *MtENOD40* has been described as highly structured and not associated to polysomes [56,59]. Yeast three-hybrid assays revealed that the structured *MtENOD40* RNA directly interacts with the constitutive RNA Binding Protein 1 (MtRBP1), a close homolog of lncRNA-interacting AtNSRs [60], located in the nuclear speckles where the splicing machinery is also hosted [61]. During nodulation, MtRBP1 is exported by *MtENOD40* to cytoplasmic granules. Hence, while *ENOD40*-encoded peptides are likely involved in sugar metabolism, the highly structured *ENOD40* RNA contributes to nucleocytoplasmic trafficking [61] (Figure 2).

## 5. Long Noncoding RNAs Promoting Translation

**Figure 3:**
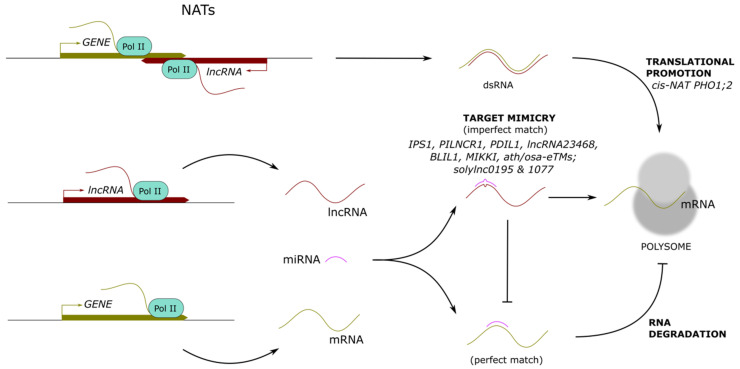
Long noncoding RNAs modulating the translation of protein-coding genes. Long noncoding (lnc) RNAs can form RNA–RNA interactions (dsRNA), promoting the shuttle to polysomes and enhancing translation. In addition, lncRNAs can act as miRNA target mimicry, titrating active miRNA abundance and boosting mRNA translation. Examples of characterized lncRNAs are indicated below each mechanism.

The translation process of mature mRNAs into proteins can be divided into four phases, namely initiation, elongation, termination, and ribosome recycling. The regulation of translation, so-called translational control, is a mechanism that allows a rapid modulation of gene expression through the activation or repression of pre-synthesized mRNA translation without requiring *de novo* transcription [62,63]. The global regulation of translation of most cellular mRNAs mainly relies on the modification of translation-initiation factors, while specific control targeting certain mRNAs likely involves regulatory protein complexes, microRNA-containing ribonucleoprotein complexes, and lncRNAs [64,65,66,67,68,69,70]. LncRNAs can be recruited to polysomes to regulate the translation of target mRNAs positively or negatively or indirectly regulate translation by sequestering miRNAs that direct the cleavage of target mRNAs (Figure 3).

### 5.1. LncRNA-mRNA Pairs into Polysomes

In rice, the *PHOSPHATE1;2* (*PHO1;2*) gene is involved in the export of phosphate to the apoplastic space of xylem vessels [71,72,73]. The complementary strand of *PHO1;2* encodes the associated *cis*-NAT *PHO1;2*. Both genes are controlled by promoters active in the vascular cylinders of roots and leaves, but while *PHO1;2* promoter is unresponsive to phosphate, *cis*-NAT *PHO1;2* promoter is strongly activated under phosphate deficiency [74]. In phosphate-deficient plants, *cis*-NAT *PHO1;2* transcripts and PHO1;2 protein amount increase, although *PHO1;2* mRNA levels remain unchanged. In addition, the downregulation or constitutive overexpression of *cis*-NAT *PHO1;2* leads, respectively, to a decrease or strong increase in PHO1;2 protein levels, whereas the level of expression and nuclear export of *PHO1;2* mRNA are not affected. Notably, the expression of *cis*-NAT *PHO1;2* is associated with the shuttle of the *PHO1;2*–*cis*-NAT *PHO1;2* sense-antisense pair towards the polysomes, supporting a role for *cis*-NAT *PHO1;2* in the promotion of *PHO1;2* translation through polysomal recruitment, to regulate phosphate homeostasis [74].

More recently, global analyses of polysome-associated RNAs and ribosome footprints in *A. thaliana* led to the identification of novel lncRNAs controlling cognate mRNA translation [70,75]. Under phosphate deficiency, five ribosome associated *cis*-NATs showed an induction correlated with the enhanced translation of their cognate sense transcripts, including two ATP BINDING CASSETTE SUBFAMILY G transporters (ABCG2 and ABCG20) and a POLLEN-SPECIFIC RECEPTOR-LIKE KINASE 7 (PRK7) family member, associated with nutrient uptake, lateral root formation, and root cell elongation, respectively [70]. In addition, five *trans*-NATs showed a positive correlation between their expression and their target mRNA levels, and the expression of four *trans*-NATs was found to correlate with a change in target mRNA polysome association under low phosphate conditions [75].

### 5.2. LncRNAs as Target Mimics for miRNAs

MiRNAs are ncRNAs of 20–22 nucleotides that play key regulatory roles in various biological processes in plants [76]. They are processed by Dicer-like proteins from imperfectly paired stem-loop precursors and repress gene expression by directing the cleavage or the translational arrest of target mRNAs [77,78,79]. Some lncRNAs with highly similar target sites as miRNA targets (miRNA recognition elements) can act as inhibitors of miRNA activity. They function as competing endogenous RNAs (ceRNAs), binding to miRNAs with imperfect base complementarity and blocking their interaction with authentic targets [80,81]. This regulatory mechanism is known as “target mimicry”. In plants, ceRNAs are named “target mimics” (TMs), also referred to as miRNA sponges or miRNA decoys in mammals.

In *A. thaliana*, the lncRNA *INDUCED BY PHOSPHATE STARVATION 1* (*IPS1*) is a functional endogenous target mimic (eTM) of miR399 involved in inorganic phosphate (Pi) homeostasis [82]. The Pi starvation-responsive AtmiR399 directs the cleavage of the mRNA *AtPHO2* (*Phosphate 2*), encoding an E2 ubiquitin conjugase-related protein, which negatively regulates Pi remobilization and Pi content in shoots. The sequences of the mRNA *AtPHO2* and lncRNA *IPS1* contain a similar motif of 23 nucleotides complementary to AtmiR399. However, in contrast to *AtPHO2*, *IPS1* pairing with AtmiR399 is interrupted by a mismatched loop in the expected AtmiR399 cleavage site, which prevents its degradation. When *IPS1* sequesters AtmiR399, the authentic AtmiR399-target *AtPHO2* is accumulated, leading to a decrease in shoot Pi content [82]. More recently, a very similar mechanism was reported in maize. The lncRNA *PI-DEFICIENCY-INDUCED LONG NONCODING RNA 1* (*PILNCR1*) functions as an eTM for ZmmiR399, thwarting the ZmmiR399-guided posttranscriptional repression of *ZmPHO2* and favoring maize adaptation to Pi deficiency [83]. Additionally, in *Medicago truncatula*, the *PI-DEFICIENCY-INDUCED LNCRNA 1* (*PDIL1*) was reported to regulate Pi transport by inhibiting the degradation of *MtPHO2*, also acting as an eTM for MtmiR399 [84]. Another example of lncRNA functioning as eTM is the tomato *lncRNA23468* involved in the resistance to *Phytophthora infestans* [85]. Overexpression of *lncRNA23468* induces a significant decrease in miR482b accumulation and an increase in the miR482b target genes *NBS-LRR* (nucleotide binding sites-leucine-rich repeat) expression. It was thus proposed that *lncRNA23468* may decoy miR482b for targeted cleavage, thereby increasing the expression levels of *NBS-LRRs* genes, enhancing tomato resistance to *P. infestans* [85]. 

Computational analyses also led to the identification of putative eTMs originating from intergenic or noncoding genes for 20 highly conserved miRNAs in *Arabidopsis thaliana* and rice [86]. The identified TMs for miR160 (ath-eTM160-1 and osa-eTM160-3) and miR166 (ath-eTM166-1 and osa-eTM166-2) were proven to be functional target mimics, their overexpression leading to diverse altered phenotypes such as smaller and serrated leaves, spoon-shaped cotyledons, curled rosette leaves, or accelerated flowering. The effectiveness of TMs for miR156, miR159, and miR172 was also confirmed by transient agroinfiltration assay [86]. In tomato, the lncRNAs *slylnc0195* and *slylnc1077* involved in the tomato yellow leaf curl virus response were predicted to be eTMs of miR166 and miR399, respectively, and the functionality of *slylnc0195* was also validated using a transient agro-infiltration assay [87]. Recently, 407 competing endogenous (ce)RNA pairs were constructed in *A. thaliana* to identify lncRNAs involved in blue light-directed plant photomorphogenesis and acting as ceRNAs. The lncRNA *BLUE LIGHT-INDUCED LNCRNA 1* (*BLIL1*) was found to inhibit hypocotyl elongation under blue light and in response to mannitol stress by serving as a ceRNA to sequester miR167 [88].

Interestingly, the mechanism of target mimicry can be engineered and exploited to inhibit specific miRNAs via artificial miRNA TMs (aTMs) in order to establish their functionality. In *A. thaliana*, a collection of transgenic plants expressing aTMs predicted to reduce the activity of most of the miRNA families was generated, leading to morphological abnormalities in the aerial part for ~20% of the miRNAs targeted [89]. 

Finally, transposable element (TE)-derived transcripts that contain binding sites for miRNAs can also function as eTMs. In rice, the retrotransposon-derived transcript *MIKKI* (“decoy” in Korean) was identified as an eTM for miR171, known to target mRNAs encoding SCARECROW-Like (SCL) transcription factors for cleavage [90]. *MIKKI* is a TE-derived locus including *Osr29* Long Terminal Repeat (LTR) retrotransposon, and its mature transcript contains an imperfect binding site for miR171, generated by a splicing event and likely attenuating the cleavage activity of miR171. In roots, *MIKKI* transcripts bind to miR171, stabilizing *SCL* mRNAs, which play an important role in root development [90]. 

## 6. Long Noncoding RNAs Mediating Post-Translational Modifications: Impact on Chromatin Remodeling and Transcription

In mammals, several examples illustrate the action of lncRNAs in protein post-translational modifications. By bringing together target proteins and specific kinases, phosphatases, or ubiquitin-ligases, lncRNAs can regulate post-translational modifications that will modulate the activity of enzymes [91,92], the stability of proteins [93], or their sub-cellular localization [94]. Intriguingly, the only known post-translational modifications modulated by plant lncRNAs are related to histones, thus affecting the epigenetic profile of target genes and their transcriptional status. The epigenetic regulation of gene expression by lncRNAs has been recently reviewed [17]. Here, we focus on the histone post-translational modifications modulated by lncRNAs in plants.

**Figure 4:**
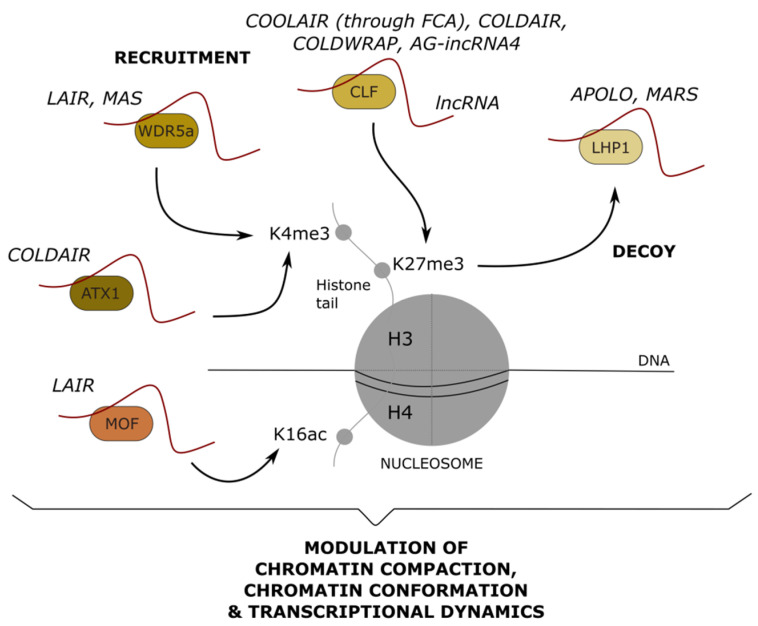
Long noncoding RNAs modulating post-translational modifications of histone proteins. Long noncoding (lnc) RNAs can recruit or decoy nuclear protein complexes that modify histone tails. H3K4 trimethylation (me3) can be modulated by the lncRNA-mediated recruitment of WDR5a (COMPASS-like complex) or ATX1 (Trithorax). H3K27 trimethylation (me3) can be modulated by the lncRNA-mediated recruitment of CLF (PRC2) or the decoy of LHP1 (PRC1). Finally, H4K16 acetylation (ac) can be modulated by the recruitment of MOF. The molecular output of histone post-translational modifications on chromatin and transcription is indicated below. Examples of characterized lncRNAs are indicated above each chromatin-related player.

Polycomb Group (PcG) proteins are critical regulators of gene expression, essential for development in many organisms. They form complexes that modify post-translationally histones tails of target genes. In plants, the histone H3K27 trimethyltransferase CURLY LEAF (CLF) functions as a catalytic subunit of the Polycomb Repressive Complex 2 (PRC2) complex [95,96,97]. H3K27me3 then assists to recruit the PRC1-like components LIKE HETEROCHROMATIN PROTEIN 1 (LHP1) and AtRING1 [98]. Additionally, the Trithorax H3K4 methyltransferase ARABIDOPSIS TRITHORAX-LIKE PROTEIN 1 (ATX1) mediates the establishment of H3K4me3 [99]. Interestingly, various lncRNAs have been associated with the post-translational modifications of histones at target loci, mediated by the recruitment or removal of PcG and Trithorax proteins (Figure 4).

In *A. thaliana*, the *FLOWERING LOCUS C* (*FLC*) gene encodes a MADS-box-containing transcription factor (TF) that acts as a critical repressor of flowering [100]. *FLC* transcription is antagonistically regulated not only by the active histone modifications H3K4me3 and H3K36me3 but also by the repressive histone modification H3K27me3 [101]. Upon transition to flowering, H3K4me3 is removed, while H3K27me3 is deposited, leading to a decrease in *FLC* expression [102]. Remarkably, *FLC* transcriptional regulation depends on *cis*-acting lncRNAs, including *COOLAIR*, *COLD ASSISTED INTRONIC NONCODING RNA* (*COLDAIR*), and *COLDWRAP* [103,104,105,106]. *COOLAIR* is a set of antisense transcripts physically associated with the *FLC* locus, linked to the synchronized replacement of H3K36 methylation with H3K27me3 during the early stages of vernalization, independent of Polycomb complexes [107]. *COOLAIR* directly binds to the RNA binding protein FLOWERING CONTROL LOCUS A (FCA), which further interacts with the PRC2 component CLF. This allows the recruitment of CLF at *FLC* for H3K27me3 deposition [108]. *COLDAIR* is transcribed from the first intron of *FLC* and cooperates with *COLDWRAP*, derived from *FLC* proximal promoter, to facilitate the establishment of H3K27me3 during the late stage of vernalization, through the formation of a repressive intragenic chromatin loop that retains CLF at the *FLC* promoter [104,105]. Strikingly, ectopic overexpression of *COLDAIR* suppresses H3K27me3 and induces H3K4me3 at the *FLC* locus depending on the recruitment of ATX1 and removal of CLF, leading to enhanced *FLC* expression [109]. Remarkably, the overexpression of intronic lncRNAs derived from several other H3K27me3-enriched MADS-box genes also led to the activation of their corresponding genes by suppressing H3K27me3 and promoting H3K4me3 deposition [109]. The NAT-lncRNA *MADS AFFECTING FLOWERING4* (*MAS*), transcribed from the *MADS AFFECTING FLOWERING 4* (*MAF4*) locus, is also involved in the regulation of flowering [110]. *MAS* is induced by cold and activates *MAF4*, encoding a MADS-box containing TF, by interacting with WDR5a, a structural core component of a COMPASS-like H3K4 histone methylation complex. *MAS* mediates the recruitment of WDR5a to *MAF4* for H3K4me3 deposition and activation of *MAF4* [110]. In rice, the lncRNA *LRK Antisense Intergenic RNA* (*LAIR*) is transcribed from the antisense strand of its neighboring *LRK* (leucine-rich repeat receptor kinase) gene cluster and can interact with OsWDR5 as well as with the histone H4K16 acetyltransferase OsMOF [111]. *LAIR* overexpression is associated with higher H3K4me3 and H4K16ac levels at the *LRK1* chromatin region and with the upregulation of *RLK1*, leading to increased grain yield [111]. AGAMOUS (AG) is another MADS TF, involved in the specification of stamens and carpels, in a tissue-specific manner [112,113,114]. The *AG* second intron encodes several ncRNAs, including *AGAMOUS INTRONIC RNA 4* (*AG-incRNA4*), which recruits CLF and represses *AG* transcription likely through the deposition of H3K27me3 [115].

In *A. thaliana*, the PRC1 protein LHP1 recognizes H3K27me3 deposition and ensures the spreading of this repressive mark, controlling global genome topology [116]. In response to auxin, the lncRNA *AUXIN REGULATED PROMOTER LOOP* (*APOLO*) is transcribed from the promoter region of its neighboring gene *PINOID,* a key regulator of polar auxin transport, and interacts with LHP1 in vivo [117]. *APOLO* recognizes a subset of auxin-related genes in *trans*, through sequence complementarity and DNA–RNA hybrid formation (R-loops). Remarkably, overexpression of *APOLO* leads to the decoy of LHP1 from common target genes over the genome, and is associated with a decrease in H3K27me3 deposition as well as with modifications of chromatin conformation [118]. Similarly, the lncRNA *MARNERAL SILENCING* (*MARS*) is transcribed in response to abscisic acid (ABA) from the marneral cluster, which includes the marneral synthase *MRN1* gene and the two P450 cytochrome-encoding genes *CYP705A12* and *CYP71A16* [119]. *MARS* over-accumulation is associated with the decoy of LHP1 and a decrease in H3K27me3 distribution throughout the marneral cluster. Loss of H3K27me3 likely allows the formation of a chromatin loop bringing together an enhancer element enriched in ABA-related TF binding sites and *MRN1* proximal promoter, resulting in the transcriptional activation of *MRN1* and a delay in seed germination [119].

## 7. Conclusions and Future Perspectives

Compelling evidence supports the involvement of lncRNAs in diverse and numerous aspects of post-transcriptional gene regulations in plants. Future research will likely shed light on the basis governing lncRNA interaction with diverse molecular partners, including DNA, proteins, or transcripts. The noncoding transcriptome has been shown to differ across ecotypes of the same species, notably in response to the environment [120]. This observation suggests that lncRNAs may be the key players in natural variation, contributing to plant adaptation during evolution. The conserved role of divergent lncRNAs across species likely depends on the presence of specific short sequences as well as on their secondary structure. Remarkably, the growing number of identified cold-responsive lncRNAs participating not only in the post-transcriptional but also in the transcriptional regulation of gene expression [121], e.g., the lncRNA *SVALKA* [122], suggests that the noncoding transcriptome is a central actor in responses to the environment. Notably, as plants cannot regulate their corporal temperature, the structure of lncRNAs and mRNAs, as well as their interactions, is most likely affected by this environmental cue. In agreement with this hypothesis, it has been recently demonstrated that the secondary structure of plant mRNAs in response to warm temperatures may modulate their translational activity, acting as thermosensor tools [123]. Similarly, structured regions in bacterial mRNAs, named RNA thermometers (RNATs), can function as thermosensors and regulate translation [124]. The advent of cutting-edge technologies, including SHAPE-seq (selective 2’-hydroxyl acylation analyzed by primer extension sequencing) to characterize RNA folding [125], will likely allow one to determine whether plant lncRNAs adopt alternative structures in response to temperature, and might function as new emerging regulators fine-tuning the protein-coding genome in response to climate change.

## Data Availability

No new data were created or analyzed in this study. Data sharing is not applicable to this article.
